# Effect of heat-clearing and dampness-eliminating Chinese medicine for high-risk cervical cancer papillomavirus infection: a systematic review and meta-analysis of randomized controlled trials

**DOI:** 10.3389/fmed.2023.1022030

**Published:** 2023-08-24

**Authors:** Shan Huang, Yuanjie Qi, Shouzhen Chen, Baochang He, Xueli Chen, Jinbang Xu

**Affiliations:** ^1^Department of Traditional Chinese Medicine, College of Clinical Medicine for Obstetrics and Gynecology and Pediatrics, Fujian Maternity and Child Health Hospital, Fujian Medical University, Fuzhou, Fujian, China; ^2^Department of Gynecology, College of Clinical Medicine for Obstetrics and Gynecology and Pediatrics, Fujian Maternity and Child Health Hospital, Fujian Medical University, Fuzhou, Fujian, China; ^3^Department of Obstetrics, College of Clinical Medicine for Obstetrics and Gynecology and Pediatrics, Fujian Maternity and Child Health Hospital, Fujian Medical University, Fuzhou, Fujian, China; ^4^Fujian Provincial Key Laboratory of Environment Factors and Cancer, Department of Epidemiology and Health Statistics, School of Public Health, Fujian Medical University, Fuzhou, Fujian, China

**Keywords:** high-risk human papillomavirus, randomized controlled trials, traditional Chinese medicine, heat-clearing and dampness-eliminating therapy, meta-analysis

## Abstract

**Background:**

Heat-clearing and dampness-eliminating Chinese medicine (HDCM) has been studied in clinical trials for cervical HPV infection for decades. However, there has been little comprehensive assessment of the strength and quality of the evidence. Therefore, this study conducted a systematic review and meta-analysis to assess the effectiveness and safety of HDCM in high-risk cervical HPV-infected patients.

**Methods:**

The research focus questions were constructed in accordance with the criteria of participants, intervention, comparison, and outcomes (PICO), and a protocol was registered in PROSPERO. Comprehensive and systematic searches and inquiries in eight electronic databases were conducted from their inception to 30th June 2022. Further, a systematic review and meta-analysis of all randomized controlled trials (RCTs) were conducted to evaluate the HDCM therapy methods.

**Results:**

A total of 12 studies were eligible for inclusion, including 1,574 patients. Data synthesis showed that the HPV clearance rate of HDCM groups was superior to both interferon and follow-up groups (RR = 1.40,95% CI:1.15, 1.71, *P* < 0.01) and (RR = 3.15, 95% CI:2.43,4.08, *P* < 0.01), respectively. HDCM was proven to exhibit greater potential in reducing HPV-DNA virus load (MD = −5.16, 95% CI: −5.91, −4.41, *P* < 0.01). The reversal rate of cervical intraepithelial neoplasia (CIN) for HDCM groups was approximately 2.8 times (RR = 2.80, 95% CI: 2.19, 3.57, *P* < 0.01), as high as the follow-up groups. Additionally, the recurrence rate of HR-HPV at the end of follow-up in this meta-analysis was reported to be lower in HDCM groups compared to follow-up groups [6.81% (16/235) and 14.65% (29/198), respectively]. The most commonly used Chinese herbal remedies were as follows: Huangbai (*Phellodendron chinense var.Glabriusculum* C.K. Schneid.), Kushen (*Sophora flavescens* Aiton), Daqingye (*Isatis indigotica* Fortune), Zicao (*Arnebia hi-spidissima* DC.), Baihuasheshecao (*Hedyotis diffusa* Spreng.), Banlangen (*Isatis tinctoria subsp.tinctoria* L.), Huzhang (*Reynoutria japonica* Houtt.), and Huangqi (*Orobanche astragali* Mouterde).

**Conclusion:**

HDCM interventions appeared to generate significant effects on enhancing the rate of HR-HPV clearance, reducing the HPV-DNA virus load, and increasing the CIN regression rate. Some active components were confirmed to be responsible for this efficacy, which deserves further exploration.

**Systematic review registration:**

https://www.crd.york.ac.uk/PROSPERO/, identifier: CRD42022333226.

## 1. Introduction

Human papillomaviruses (HPVs) are a major cause of malignancy worldwide, leading to a host of human cancers (1–3). Studies have confirmed that infection with high-risk human papillomavirus (HR-HPV) is a crucial factor for the development of cervical cancer and its precancerous cervical intraepithelial neoplasia (CIN) (4). HPV is mainly transmitted through sexual activity. According to relevant data, approximately 80% of people with normal sexual lives may be infected with HPV at some point in their lives (5), and ~90% of HPV infections can be cleared within 6–18 months after acquisition (6, 7). However, for others, the infection may persist and give rise to precancerous changes.

For decades, the HPV vaccine has contributed to the decline in cervical cancer cases, which is also related to lower mortality (8, 9). However, for persistent HR-HPV infections, there is still no desirable treatment available to medical workers. The American Society for Colposcopy and Cervical Pathology(ASCCP) guidelines recommend (10) more frequent surveillance, colposcopy, and treatment for patients at progressively elevated risk, whereas those at lower risk can defer colposcopy, undergo follow-up at longer surveillance intervals, and return to routine screening when at sufficiently low risk. Nevertheless, patients with persistent infection for 1–2 years tend to experience a higher risk of CIN than patients with initial infection (11, 12).

Chinese herbal medicine (CHM) has a long history of treating numerous human diseases in Asian countries (13). With the advantages of multiple components, multiple targets, multiple links, and multiple pathways, CHM has been proposed as a potential candidate for the treatment of HPV infection. Previous systematic reviews and meta-analyses (14, 15) have indicated that CHM improves clinical index in the treatment of cervical cancer and genital HPV infection. However, only some TCM syndromes were explored without analyzing the TCM syndrome differentiation of HPV, easily resulting in confounding bias. Based on the theory of traditional Chinese medicine (TCM), heat dampness, an etiological factor in TCM, is the main pathogenesis of HR-HPV infection. The classic TCM treatment for heat-dampness syndrome is clearing heat and eliminating dampness. In recent decades, evidence-based studies of heat-clearing and dampness-eliminating Chinese medicine (HDCM) in treating cervical HR-HPV infection have been all the rage (16–18). However, there is no systematic investigation of HDCM for HR-HPV infection. Given this, it is necessary to conduct a systematic review of the efficacy and safety of HDCM in HR-HPV infection.

## 2. Materials and methods

A systematic review was conducted according to the Preferred Reporting Items for Systematic Reviews and Meta-Analysis (PRISMA) statement. The review was registered in PROSPERO with the registration number CRD42022333226.

### 2.1. Search strategy for the identification studies

PubMed, Embase, Cochrane Library, Medline, China National Knowledge Infrastructure (CNKI), Chinese Scientific Journal Database (VIP), Wanfang Database, and Sinomed were systematically searched from inception to 30th June 2022. Search terms included the following: (“Human Papillomavirus” or “HPV Human Papillomavirus” or “HPV Human Papillomaviruses” or “Human Papillomavirus, HPV” or “Human Papillomaviruses, HPV” or “Human Papillomavirus” or “Human Papillomaviruses” or “Papillomavirus, Human” or “Papillomaviruses, Human”) and (“TCM” or “traditional Chinese medicine” or “Chinese medicinal herb” or “Chinese herbal medicine” or “decoction” or “formula” or “prescription” or “Chinese patent medicine” or “Chinese patent drug” or “Chinese herbal compound prescription”) and (“randomized controlled trial” or “controlled clinical trial” or “random” or “randomly” or “randomized” or “control” or “RCT”). The authors of the identified studies were contacted for additional information if necessary.

### 2.2. Inclusion criteria

Randomized controlled trials (RCTs) published in any language were included. The study population (P) referred to women diagnosed with HR-HPV positive cervical lesions diagnosed as a low-grade squamous intra-epithelial lesion (LSIL) or below by histopathological examination, meeting the diagnostic points formulated by ASCCP (10) and the Textbook of the 12th five-year plan of the Ministry of Health, Obstetrics, and Gynecology (People's Medical Publishing House, 8th edition, 2018, edited by Xie Xing and Kong Beihua). The interventions (I) were HDCM used alone, including both single Chinese herbs and Chinese herbal formulae such as decoction, capsule, tablet, granule, or pill. The controls (C) were interferon or follow-up groups. The primary outcomes (O) involved the rate of HR-HPV clearance, the reduction of HPV-DNA virus load, and the regression rate of CIN at different follow-up time points. The secondary outcomes covered the rate of recurrence and adverse events.

### 2.3. Exclusion criteria

(1) Cervical lesions diagnosed as a high-grade squamous intraepithelial lesion (HSIL) or above by histopathological examination; (2) HDCM combined with acupuncture, TCM fumigation therapy, or other Chinese medicines; (3) duplicate literature; (4) animal experiments; (5) research data exposed serious errors or the full text was not available.

### 2.4. Data selection

Two authors (HS and QYJ) independently extracted the following:

Data: the first author, publication year, characteristics of participants, intervention methods, components of HDCM, type of HPV, duration of treatment, follow-up time, and outcome measures. Any discrepancy was resolved by consultation among the reviewers or discussion with a third author (Jinbang Xu).

### 2.5. Quality assessment

The Cochrane Risk of Bias Tool (19) was adopted to evaluate the quality of trials, focusing on seven aspects: (1) random sequence generation; (2) allocation concealment; (3) blinding of participants and personnel; (4) blinding of outcome assessment; (5) incomplete outcome data; (6) selective outcome reporting; and (7) other sources of bias. The evaluation criteria for each item were judged as “low risk of bias”, “unclear risk of bias”, and “high risk of bias”.

### 2.6. Data analysis

Data analysis was completed using Review Manager 5.3. Risk ratio (RR) and mean difference (MD) with their 95% confidence intervals (CI) were employed for dichotomous outcomes and continuous outcomes separately. Heterogeneity was measured by *I*^2^ statistics. Specifically, a fixed-effect (FE) model was utilized if *I*^2^ < 50%. Otherwise, the random-effect (RE) model was used. A *p* ≤ 0.05 was considered statistically significant. Publication bias was explored by means of funnel-plot analysis.

The sources of heterogeneity were analyzed. The robustness of the results was tested by conducting the sensitivity analysis with the exclusion of studies with unclear random sequence generation. The effect of HDCM with different treatment durations was analyzed separately with subgroup analysis.

## 3. Results

### 3.1. Literature screening

**Figure 1:**
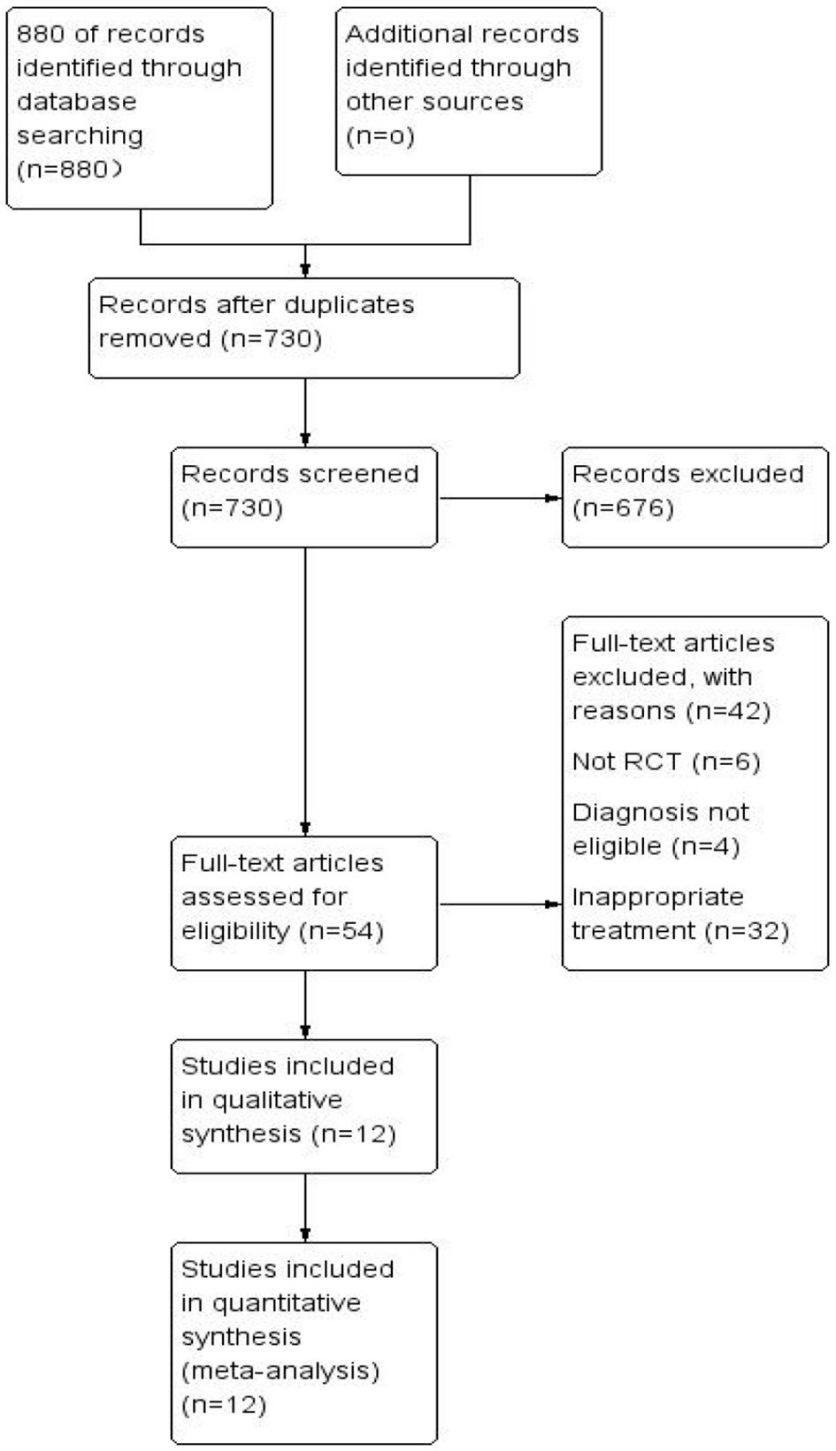
Flow diagram of included RCTs identified for meta-analysis.

A total of 880 records (PubMed [*n* = 72], Cochrane Library [*n* = 18], Medline [*n* = 23], Embase [*n* = 1], CNKI [*n* = 627], Sinomed [*n* = 101], VIP [*n* = 10], and Wanfang Data [*n* = 28]) were retrieved, with 150 duplicates removed. After reviewing the titles and abstracts, 676 records were excluded, leaving 54 records remaining. 12 RCTs were finally included after full text screening (Figure 1) (20–31): 1 in English and 11 in Chinese.

### 3.2. Characteristics of included studies

The enrolled studies published between 2011 and 2021 in China were utilized, including a total of 1,574 patients. Among them, seven studies (20–24, 27, 30) compared the treatment effect of HDCM with interferon, and six (20, 26, 28–31) focused on the comparison of follow-up. Nine studies applied vaginal preparations of HDCM, and three studies (20, 27, 28) used oral HDCM. Only one study (20) reported specific HR-HPV types. The follow-up duration ranged from 1 to 12 months. Table 1 presents the characteristics of the included studies.

**Table 1:** Characteristics of the included studies.

**References**	**Participant**		**Type of HPV**	**Intervention**			**Follow ups (months)**	**Outcome measures**
	**(** ***N*** **)**	**Age**		**H**	**I/F**	**Treatment duration (H/I)**		
Li et al. (21)	H:50 I:50	H:33.06 ± 8.51 I:33.92 ± 8.42	NR	Gongjingkang Vaginal gel, 2.0 g, qod	Interferon α-2βVaginal gel, 2.0g qod	Three menstrual cycles,10 times per cycle	3	
Lin and Ban (22)	H:300 I:300	H:28.05 ± 7.01 I:27.46 ± 6.28	NR	Gongyanxiao Vaginal Powder, N/A, qod	Interferon α-2βSuppository, 1 pill, qod	Two menstrual cycles, nine times per cycle	2	,
Zhao et al. (23)	H:40 I:40	18-60	NR	Kushen Vaginal gel, 1.0g, qd	Interferon α-2βVaginal gel, 1.0g, qd	Three menstrual cycles, stop using the drug during menstruation	3	
Chen et al. (24)	H:129 I:59	25-64	NR	Paiteling external lotion, 1–2ml, qd × 3d	Interferon α-2βSuppository, 1 pill, qd	H:One course of treatment for 7 days, for a total of 5 courses I:Three menstrual cycles,10 times per cycle	2,3	
Li (25)	H:30 I:30	H:33.28 ± 6.14 I:33.54 ± 6.05	NR	Sanhuang Vaginal Powder, 4–6 g, three times a week	Interferon α-2βSuppository, 100000IU, qod	Three menstrual cycles, stop using the drug during menstruation	1	,
Wen (26)	H:35 F:35	H:35.4 ± 6.51 F:34.00 ± 6.23	NR	Chinese compound herb; Vaginal Powder, 3g, qod	Follow-up	Ensure 30 times of medication, complete within 3 months, stop medication during menstrual period	4, 12	,
Wang (27)	H:32 I:32	H:37.52 ± 3.87 I:37.61 ± 3.56	NR	Wuweixiaodu Decoction, po, bid	Interferon α-2βVaginal gel, N/A, qod	Three menstrual cycles, stop using the drug during menstruation	1	,,
Li et al. (28)	H:30 F:30	H:35.52 ± 2.56 F:34.64 ± 2.89	NR	Yihuang Decoction, po, bid	Follow-up	Three menstrual cycles,14 days per cycle	3, 6	
Xiao et al. (29)	H:35 F:35	H:34.2 ± 7.9 F:33.0 ± 7.0	NR	Youdujing external lotion, 100ml, twice a week; Youdujing cream, twice a week	Follow-up	Three menstrual cycles, used the drug 19 times in total	3	,
Xu et al. (30)	H:50 F:50	20-53	NR	Erhuang Vaginal Powder,N/A,qod	Follow-up	One course of treatment for seven times, for a total of three courses	3, 6, 9	,,
Xu et al. (31)	H:45 I:45; F:45	32.3 ± 5.7	HPV16/18/58	TCM Ermiao Granules, po, bid	C:Interferon α-2βVaginal gel, 1.0g, qod; P: Follow-up	H:Three menstrual cycles,14 times per cycle; I:Six menstrual cycles	6	,
Xiao et al. (32)	H:36 F:11	H:33.08 ± 7.73 F:34.18 ± 7.86	NR	Youdujing external lotion, 100 ml, twice a week; Youdujing cream, twice a week	Follow-up	Three menstrual cycles, used the drug 19 times in total	3, 6	,

H, HDCM group: I, interferon group; F, follow-up group; rate of hr-HPV clearance; reduction of HPV-DNA virus load; reversal rate of CIN; rate of recurrence; adverse events.

### 3.3. Risk of bias assessment

**Figure 2:**
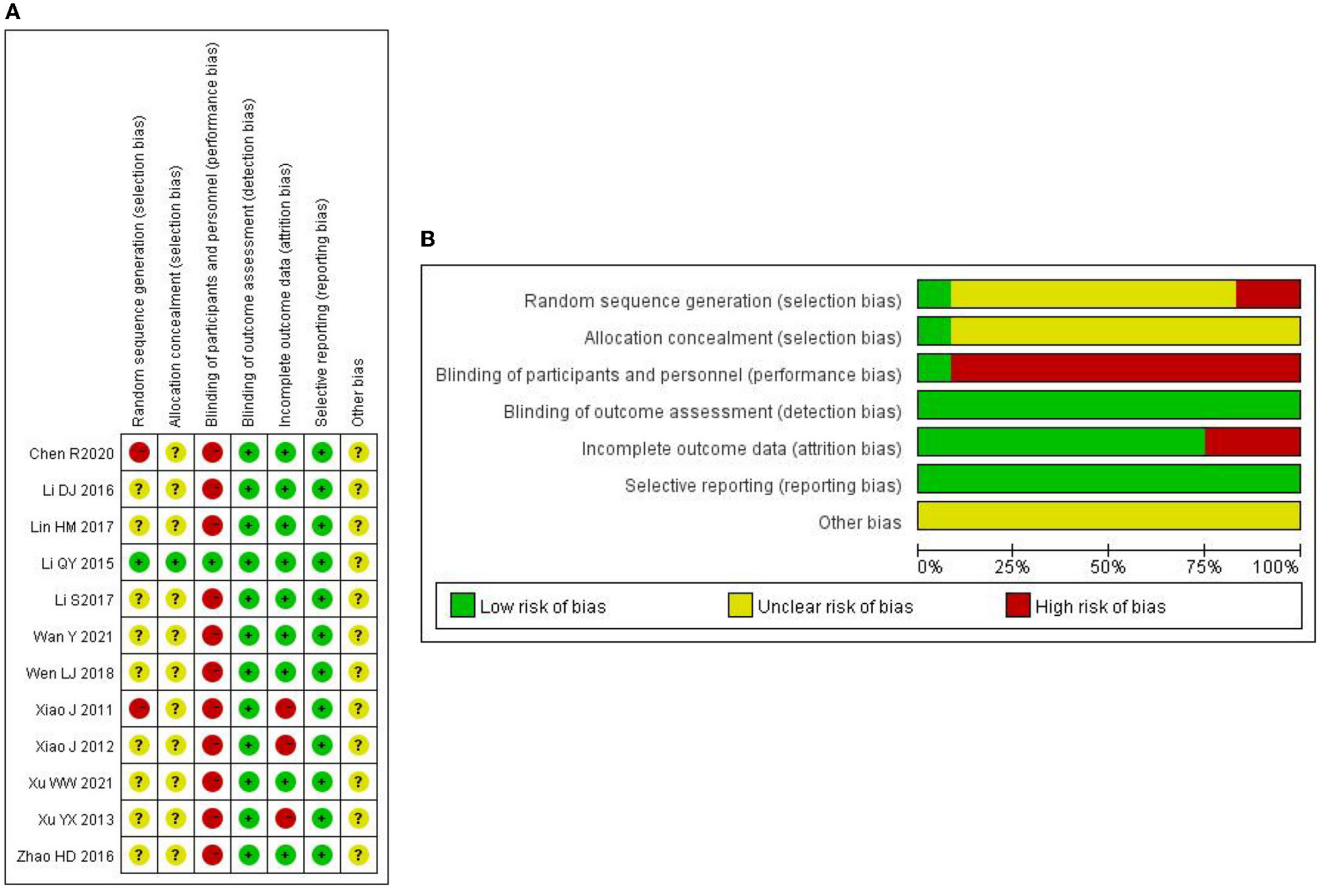
**(A)** Risk of bias assessment of randomized controlled trials using Cochrane risk of bias tool**. (B)** Risk of bias assessment of randomized controlled trials using the Cochrane risk of bias tool.

Only one study clearly reported random allocation sequence and allocation concealment (21). Two trials (24, 31) were marked as “high risk” since random design was not mentioned. The other eight trials were marked as “unclear risk” because they failed to report details on how to randomize. Apart from one trial (21), none of the other 11 trials elaborated on whether the double-blind mechanism for participants and personnel was implemented or not. Considering the objective outcome indicators used in all of these studies, researchers did not alter the results, and detection bias was identified as “low-risk.” Three trials (23, 29, 31) reported missing data. In addition, all studies reported all outcomes mentioned in part on methods, and selective reporting bias was assessed as “low-risk.” Other biases were defined as “unclear risk” by reason of the limited information provided in related reports. Figure 2 provides a summary of the risks of bias.

### 3.4. Outcomes measures

#### 3.4.1. The rate of HPV clearance

##### 3.4.1.1. HDCM treatment vs. interferon

**Figure 3:**
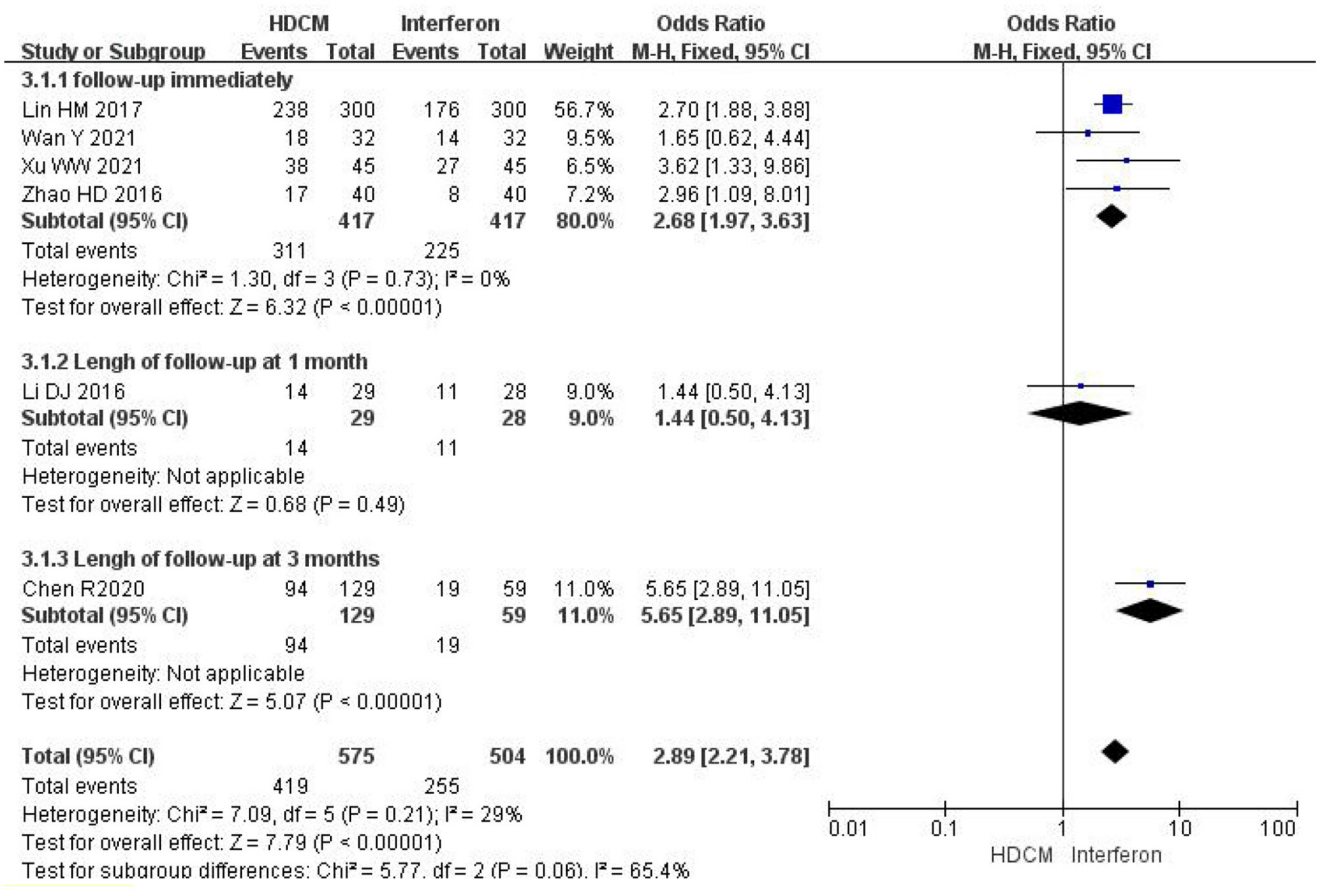
Rate of HR-HPV clearance (HDCM treatment vs. interferon). Events show the number of patients with negative HPV in each group.

Seven trials (20–25, 27) (*n* = 1,179) reported changes in HPV clearance rate before and after treatment. Due to the presence of significant heterogeneity (*I*^2^ = 54%), sensitivity analysis was conducted, proving that Li (21) was the main source of high heterogeneity. Considering that the genesis of high heterogeneity lurked in clinical heterogeneity among the included studies, this study eliminated the study Li (21) and conducted subgroup analyses to evaluate HR-HPV clearance rates at different follow-up periods. The results indicated that HDCM groups significantly improved the HPV clearance rate (RR = 2.89, 95% CI: 2.21, 3.78, *P* < 0.01). Subgroup meta-analysis was conducted according to different follow-up periods after therapy (immediately, 1 month, or 3 months). Since the treatment cycles of HDCM used in these studies included in this meta-analysis were not necessarily the same, the term “follow-up immediately” was defined as the corresponding examination immediately after the end of the treatment cycle to evaluate the efficacy. The results demonstrated no difference between HDCM and interferon groups at one-month follow-up (RR=1.4495% CI:0.50, 4.13, *P* = 0.49). Nevertheless, the effect of HDCM on promoting HPV clearance rates was significantly strengthened in other subgroups (Figure 3).

##### 3.4.1.2. HDCM treatment vs. follow-up

**Figure 4:**
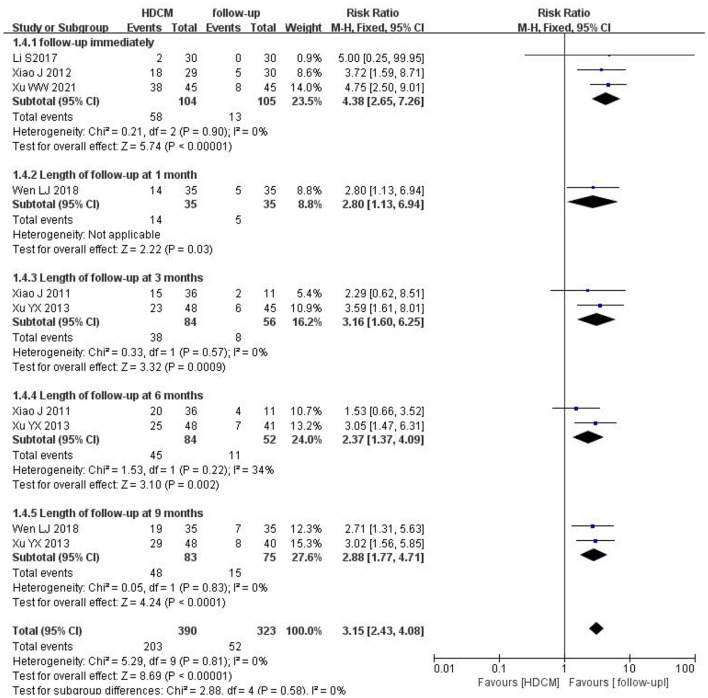
Rate of HPV clearance (HDCM treatment vs. follow-up). Events show the number of patients with negative HPV in each group.

Six trials (20, 26, 28–31) (*n* = 419) provided the HPV clearance rate. Since no significant heterogeneity was found across these studies (*I*^2^ = 0%), a fixed effect model was selected. The result yielded a pooled RR of 3.15 (95%CI: 2.43, 4.08; *P* < 0.01), as shown in Figure 4. HDCM's effectiveness was significantly higher. There was no difference among subgroup analyses at different follow-up periods (interaction *P* = 0.58).

#### 3.4.2. The reduction of HPV-DNA virus load

**Figure 5:**
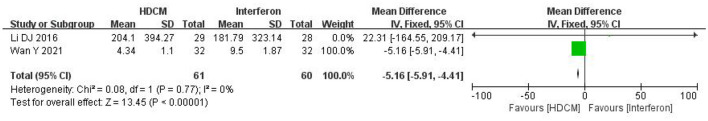
Reduction of the HPV-DNA virus load.

Two studies (25, 27) reported changes in the HPV-DNA virus load (*n* = 121). The fixed effect model was employed (*I*^2^ = 0%), and the meta-analysis showed a significant difference between the HDCM and interferon groups (MD = −5.16, 95%CI: −5.91, −4.41; *P* < 0.01). HDCM treatment induced a reduction in HPV-DNA virus load (Figure 5).

#### 3.4.3. The reversal rate of CIN

##### 3.4.3.1. HDCM treatment vs. follow-up

**Figure 6:**
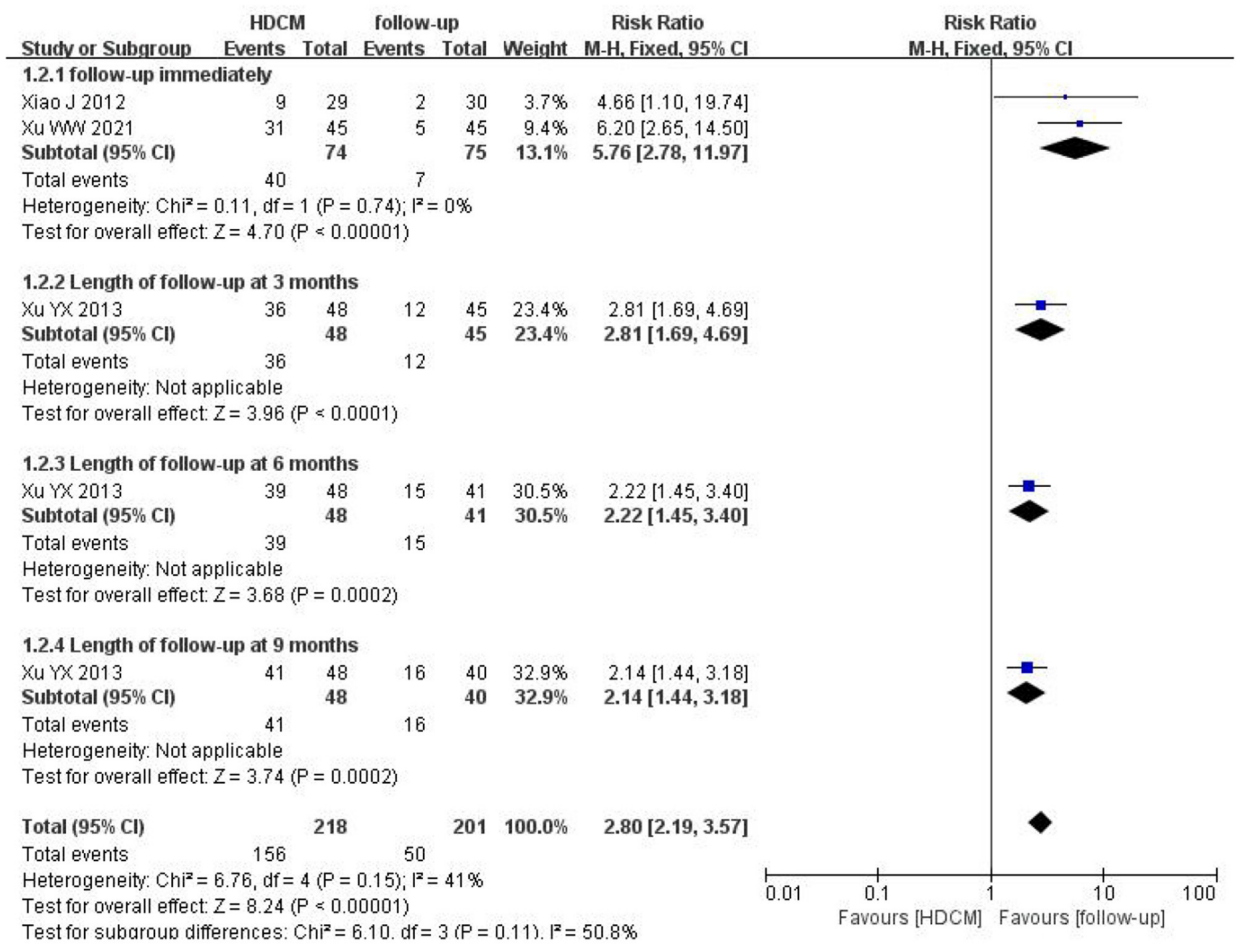
Reversal rate of CIN (HDCM treatment vs. follow-up). Events show the number of patients with a positive reversal rate of CIN.

In this study, three trials (20, 29, 30) (*n* = 242) comparing HDCM treatment with follow-up reported a CIN reversal rate. The result indicated that HDCM treatment led to a higher reversal rate of CIN (RR = 2.80; 95% CI: 2.19, 3.57; *P* < 0.01). Four subgroup analyses were conducted according to different follow-up periods (immediately, 3 months, 6 months, or 9 months), suggesting no difference (interaction *P* = 0.11) (Figure 6).

##### 3.4.3.2. HDCM treatment vs. interferon

One trial (20) (*n* = 90) reported a CIN reversal rate after treatment. The study result was statistically significant between HDCM treatment [69% (31/45)] and interferon groups [42% (19/45)], indicating the effectiveness of HDCM in inhibiting the progress of CIN.

#### 3.4.4. The rate of recurrence

Only one trial (22) (*n* = 600) investigated the recurrent rate of HPV infection. The result indicated that the recurrence rate of the HDCM group [6.81% (16/235)] was significantly lower than that of the interferon group [14.65% (29/198)] after a 1-year follow-up.

#### 3.4.5. Adverse events

**Figure 7:**
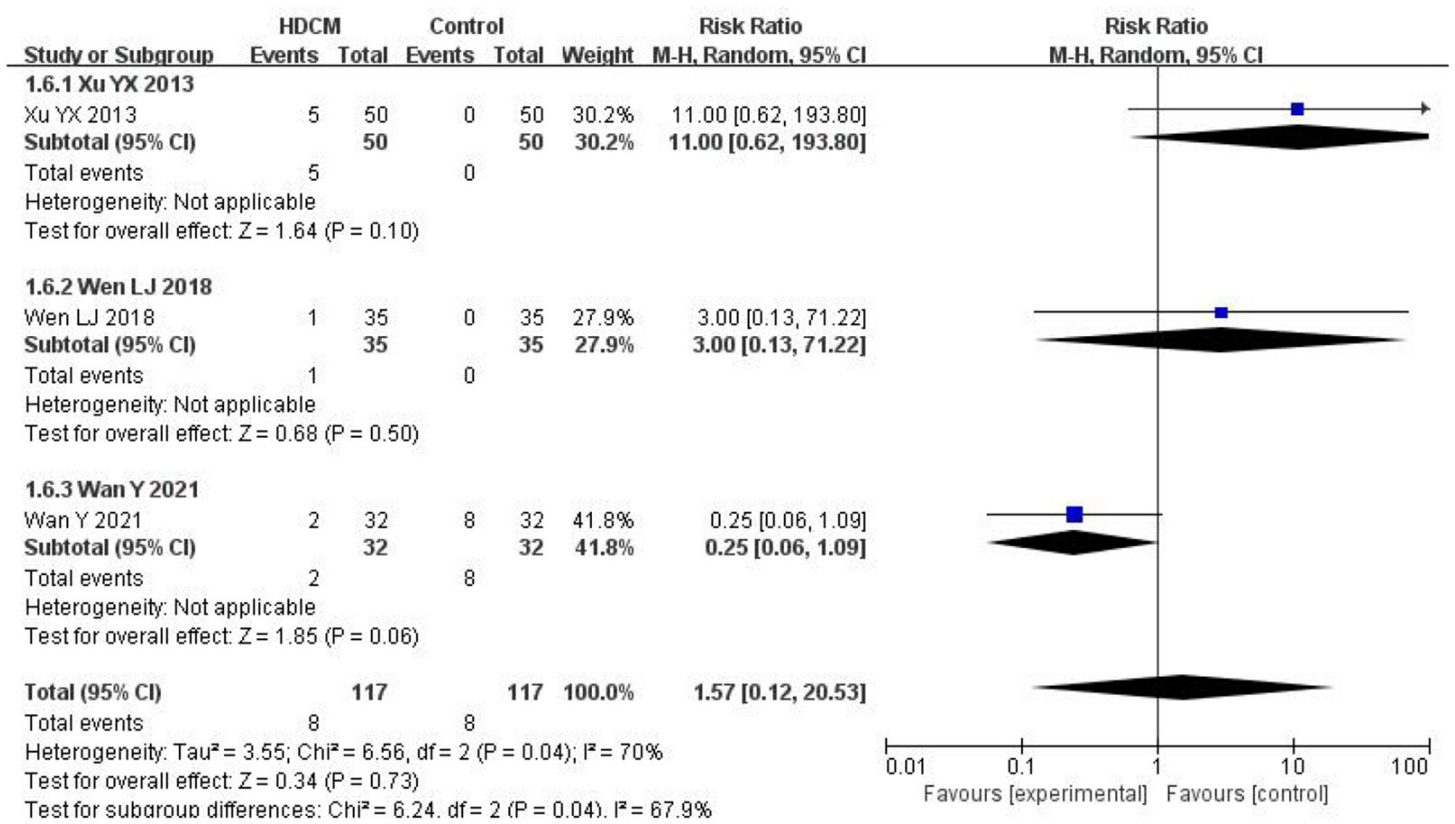
Adverse events. Events show the number of patients who suffered from adverse events in each group.

These studies (26, 27, 30) observed adverse events, while one study (25) stated that all patients did not produce adverse reactions during treatment. No adverse events were mentioned in the remaining studies. The safety of HDCM was evaluated by calculating the incidence of adverse events (Figure 7). The result showed that the incidence of adverse events between HDCM and control groups was not significantly different (RR = 1.57, 95% CI:0.12, 20.53, *P* = 0.73), indicating the safety of HDCM.

#### 3.4.6. High-frequency Chinese herbs

Table 2 describes the Chinese herbal medicine in the included studies. By statistically calculating the most commonly used herbal medicine in HDCM in the study, Chinese herbs were categorized from high to low according to their frequency of occurrence. As shown in Table 3, the top eight included: Huangbai (*Phellodendron chinense var. Glabriusculum* C.K.Schneid.) (7, 10.1%), Kushen (*Sophora flavescens* Aiton) (5, 7.2%), Daqingye (*Isatis indigotica* Fortune) (3, 4.3%), Zicao (*Arnebia hispidissima* DC.) (3, 4.3%), Baihuasheshecao (*Hedyotis diffusa* Spreng) (3, 4.3%), Banlangen (*Isatis tinctoria subsp.tinctoria* L.) (3, 4.3%), Huzhang (*Reynoutria japonica* Houtt) (3, 4.3%), and Huangqi (*Orobanche astragali* Mouterde) (3, 4.3%) [http://www.worldfloraonline.org (accessed 24th January)].

**Table 2:** CHM in the included studies.

**References**	**Formula**	**Composition of formula**
Li et al. (21)	Gongjingkang gel	Liaoqiao (Forsythia suspensa Vahl), Kushen (*Sophora flavescens* Aiton), Baiji (*Bletilla scopulorum* Schltr.), Ezhu [*Curcuma zedoaria* (Christm.) Roscoe], Huangqi (*Orobanche astragali* Mouterde)
Lin and Ban (22)	Gongyanxiao powder	Shechuangzi (*Cnidium monnieri* Cusson), baizhi [*Angelica dahurica* (Hoffm.) Benth. and Hook.f.ex Franch. and Sav.], Huangqi [Astragalus mongholicus f. albiflorus (Y.N.Lee) M.Kim], Qingdai (*Indigofera tinctoria subsp.tinctoria* L.), zhusha [*Draco cinnabari* (Balf.f.) Kuntze], Xuejie (*Calamus draco* Willd.)
Zhao et al. (23)	Kushen gel	Kushen (*Sophora flavescens* Aiton)
Chen et al. (24)	paiteling lotion	Daqingye (*Isatis indigotica* Fortune), Jingyinhua (*Lonicera praeflorens var.japonica* H.Hara), Kushen (Sophora flavescens Aiton), Yadanzi (*Brucea javanica* Merr.), Huzhang (*Reynoutria japonica* Houtt.), Fengfang (*Alpinia nidus-vespae* A.Raynal and J.Raynal)
Li (25)	Sanhuang Powder	Dahuang (R*heum officinale* Baill.), Huangqin (*Scutellaria baicalensis* Georgi), Huanglian (*Coptis teeta var.chinensis* (Franch.)Finet&Gagnep.), Huangbai (*Phellodendron chinense var.glabriusculum* C.K.Schneid.)
Wen (26)	Chinese compound herb Powder	Zicao (*Arnebia hispidissima* DC.), Huangbai (Phellodendron chinense var.glabriusculum C.K.Schneid.), Ezhu (Curcuma zedoaria (Christm.)Roscoe), Bingpian (Hydnophytum borneanum Becc.)
Wang (27)	Wuweixiaodu Decoction	Jingyinhua (Lonicera praeflorens var.japonica H.Hara) 15 g, Yejuhua (Chrysanthemum indicum L.) 15 g, Zibeitiankui (*Begonia fimbristipula* Hance) 15 g, Pugongying (*Taraxacum sect. Erythrocarpa* Hand.-Mazz.) 15 g, Zihuadiding (*Campanula violae* Pers.) 15 g, Tufuling (*Smilax ovalifolia* Roxb. ex D.Don) 30 g, Baihuasheshecao (Hedyotis diffusa Spreng.) 30 g
Li et al. (28)	Yihuang Decoction	Huangbai (*Phellodendron chinense var.glabriusculum* C.K.Schneid.) 15 g, Shanyao (*Dioscorea oppositifolia var.linnaei* Prain & Burkill) 30 g, Qianshi (*Euryale ferox* Salisb.) 30 g, Cheqianzi (*Plantago asiatica* L.) 10 g, Huangqi (Orobanche astragali Mouterde) 20 g, Baizhu (*Atractylodes macrocephala* Koidz.) 10 g, Danshen (*Codonopsis pilosula* Nannf.) 15 g, Danshen (Salvia miltiorrhiza var. miltiorrhiza) 15 g
Xiao et al. (29)	Youdujing external lotion	Zicao (Arnebia hispidissima DC.), Huzhang (Reynoutria japonica Houtt.), Daqingye (Isatis indigotica Fortune), Banlangen (Isatis tinctoria subsp. tinctoria L.), Kushen (Sophora flavescens Aiton), Huangbai (Phellodendron chinense var.glabriusculum C.K.Schneid.)
Xu et al. (30)	Erhuang Powder	Xionghuang (Realgar), Huanglian (*Coptis teeta var.chinensis* (Franch.) Finet & Gagnep.)
Xu et al. (31)	TCM Ermiao Granules	Canzhu (*Atractylodes* DC.) 10 g, Huangbai (Phellodendron chinense var.glabriusculum C.K.Schneid.) 10 g, Yiyiren (Coix lacryma-jobi var.lacryma-jobi) 30 g, Chonglou (*Macromitrium paridis* Besch.) 10 g, Banlangen (Isatis tinctoria subsp.tinctoria L.) 10 g, (Panax anemone) 30 g, Tufuling (Smilax ovalifolia Roxb. ex D.Don) 10 g
Xiao et al. (32)	Youdujing external lotion	Zicao (Arnebia hispidissima DC.), Huzhang (Reynoutria japonica Houtt.), Daqingye (Isatis indigotica Fortune), Banlangen (Isatis tinctoria subsp. tinctoria L.), Kushen (Sophora flavescens Aiton), Huangbai (Phellodendron chinense var.glabriusculum C.K.Schneid.)

**Table 3:** The most commonly used Chinese herbs.

**Chinese name**	**English name**	**Counts**	**Frequency (%)**
Huangbai	*Phellodendron chinense var.glabriusculum* C.K.Schneid.	7	10.1
Kushen	*Sophora flavescens* Aiton	5	7.2
Daqingye	*Isatis indigotica* Fortune	3	4.3
Zicao	*Arnebia hispidissima* DC.	3	4.3
Baihuasheshecao	*Hedyotis diffusa* Spreng.	3	4.3
Banlangen	*Isatis tinctoria subsp. tinctoria* L.	3	4.3
Huzhang	*Reynoutria japonica* Houtt.	3	4.3
Huangqi	*Orobanche astragali* Mouterde	3	4.3

### 3.5. Sensitivity analysis

As for the sensitivity analysis, each study in the meta-analysis was screened to reflect the impact of individual data on changes in heterogeneity. Li (21) was the main source of high heterogeneity. The heterogeneity may arise from the following aspects: (1) The HDCM components, as well as the dosing scheme of intervention measures among studies, are far removed from each other; (2) Chinese medicine usually takes a period of time to produce curative effects; (3) There are differences in HR-HPV subtypes and course of disease among participants in the study, which may give rise to higher heterogeneity. However, all of the results from the studies showed the advantages of HDCM treatment.

### 3.6. Publication bias

Publication bias analysis was investigated for each group in this study, respectively. The obtained funnel plot was asymmetric, indicating a mild publication bias (Supplementary Figures).

## 4. Discussion

### 4.1. Summary of evidence

HR-HPV infection is the leading cause of cervical cancer, which may trigger the immune escape of cervical cells, making them enter the immortalized state (32). To our knowledge, this study was the first to conduct a systematic review and meta-analysis of published RCTs to assess the efficacy of HDCM in treating cervical HR-HPV infection. It was revealed that HDCM could significantly enhance the clearance rate of HR-HPV, reduce HPV-DNA virus load, and increase the regression rate of CIN. The pooled analysis indicated that the HPV clearance rate of HDCM groups was 1.4-fold higher relative to interferon groups and 3.2-fold higher relative to follow-up groups. In terms of remission of CIN and HPV-DNA virus load, significantly greater anti-malignancy efficacy was detected in HDCM groups than in follow-up groups (RR = 2.80,95% CI:2.19, 3.57, *P* < 0.01), as well as evident antiviral efficacy (MD=-5.16, 95% CI: −5.91, −4.41, *P* < 0.01). Similarly, at the end of follow-up, the recurrence rate of HR-HPV in HDCM groups was reported to be lower than that in follow-up groups. Moreover, only mild and transient adverse events were reported, further suggesting the safety of HDCM.

Traditional Chinese medicine, which has been developed for thousands of years in China, is one of the most vital complementary and alternative medicines. There is a large body of evidence that suggests that HDCM can exert a positive effect on treating HPV infection. The *Phellodendron chinense* C.K.Schneid. Ketone from *Phellodendron chinense var.Glabriu-sculum* C.K.Schneid. can increase the expression time of the mitogen-activated protein kinase phosphatase-1(MKP-1) protein by reducing the transcription and translation levels of NO, IL-6, IL-1β, and other inflammatory factors while stabilizing the mRNA of MKP-1 by significantly inhibiting p38-mediated AP-1 signaling (33). Matrine is an important ingredient of *Sophora flavescens* Aitonis, characterized by its excellent properties against tumors by accelerating cell apoptosis, inhibiting tumor cell growth and proliferation, inducing cell cycle arrest, and preventing cancer metastasis and invasion. Moreover, it can also inhibit angiogenesis, induce autophagy, reverse multidrug resistance, and inhibit cell differentiation (34). 2-Methoxy-6-acetyl-7-methyljuglone (MAM), a natural naphthoquinone found in *Reynoutria japonica* Houtt., is proven to be effective against the progression of colon carcinoma by inducing necroptosis in cancer cells through JNK activation and mitochondrial ROS production (35). Shikonin, a naphthoquinone extracted from *Arnebia hispidissima* DC., can kill cancer cells in a necroptosis manner (36). Researchers explored that (37) *Hedyotis diffusa* Spreng. can activate murine and human antigen-presenting cells via the MAPK signaling pathway while enhancing antigen presentation in bone marrow-derived dendritic cells *in vitro*. Besides, Rutin, one of its ingredients, can trigger a strong, specific immune response against HPV-related tumors *in vivo*. Therefore, it can be reasonably speculated that the combination of HDCM can theoretically regulate the immune response, induce cell necroptosis, and enhance anti-inflammatory reactions, eventually showing a significant treatment effect on HR-HPV infection.

### 4.2. Limitations

Some limitations exposed by this study cannot be ignored. First, all of the studies involved were conducted in China, with only studies published in Chinese and English enrolled. Consequently, it is difficult to determine whether HDCM is effective for different populations worldwide. Second, with the exception of one study (20) published in the English language and available on Pubmed, most of the studies included were published in Chinese. Among the other 11 studies, two of them were available as abstracts in English with no full text, and the remaining studies were published solely in Chinese. As a result, only one of the included RCTs can be critically assessed by readers from other countries outside of China, which is one of the main shortcomings of the article.

Third, the TCM diagnosis of diseases is mainly based on the evaluation of a series of subtle variables and patient characteristics. Doctors generally judge the curative effect by collecting the characteristics of these subtle changes in patients after using drugs, which may cause a higher risk of allocation bias. Therefore, strict allocation and concealment become more important in RCTs evaluating the therapeutic effect of TCM. The descriptions of randomization, allocation concealment, and blinding methods in most of the included studies are insufficient or absent, which leads to the inability to fully and systematically evaluate the internal validity of studies. Fourth, the heterogeneous characteristics of all patients are a significant limitation of our study. Possible confounding factors included patients' age, type of HPV, course of the disease, HDCM components, and dosage regimens. Fifth, the combination, dosage form, and chemical composition of traditional Chinese medicine in the HDCM formula in each study are different, which is also one of the reasons for the heterogeneity of studies. Sixth, the follow-up time of most studies is too short to provide strong evidence for data research and analysis of the recurrence rate. Furthermore, the lack of HPV infection genotype in most studies makes it impossible for this study to obtain multiple infection data from original studies, which also makes it difficult to determine whether it is a recurrence or reinfection.

## 5. Conclusion

According to this systematic review, HDCM interventions appeared to have significant effects on enhancing the rate of HR-HPV clearance, reducing the HPV-DNA virus load, and increasing the CIN regression rate. However, considering the small sample size and poor methodological quality of the included studies, the true potential of HDCM for HPV should be validated in large-scale, multicenter, and well-designed RCTs in the future.

In addition, the efficacy of HDCM in treating HR-HPV infection was proven to be different. It has been recommended to carefully choose a suitable HDCM in clinical practice. RCTs conducted in this study can provide certain reference values for dosage and course of treatment. Moreover, seeking additional guidance from traditional Chinese medicine practitioners can also be referred to and utilized.

## Data availability statement

The original contributions presented in the study are included in the article/Supplementary material, further inquiries can be directed to the corresponding author.

## Author contributions

SH designed and conceived this study, material preparation, and data collection. YQ designed and conceived this study, including the original draft preparation. SC completed the revision process. BH conducted the formal analysis. XC performed the formal analysis. JX designed and conceived this study. Any disagreement was resolved through discussion. All authors approved the final version of the submitted manuscript.
