# Supplementary figures and images for: Effect of heat-clearing and dampness-eliminating Chinese medicine for high-risk cervical cancer papillomavirus infection: a systematic review and meta-analysis of randomized controlled trials
Source: Front Med (Lausanne). 2023 Aug 24;10:1022030. doi: 10.3389/fmed.2023.1022030 (PMC10484520; doi:10.3389/fmed.2023.1022030)

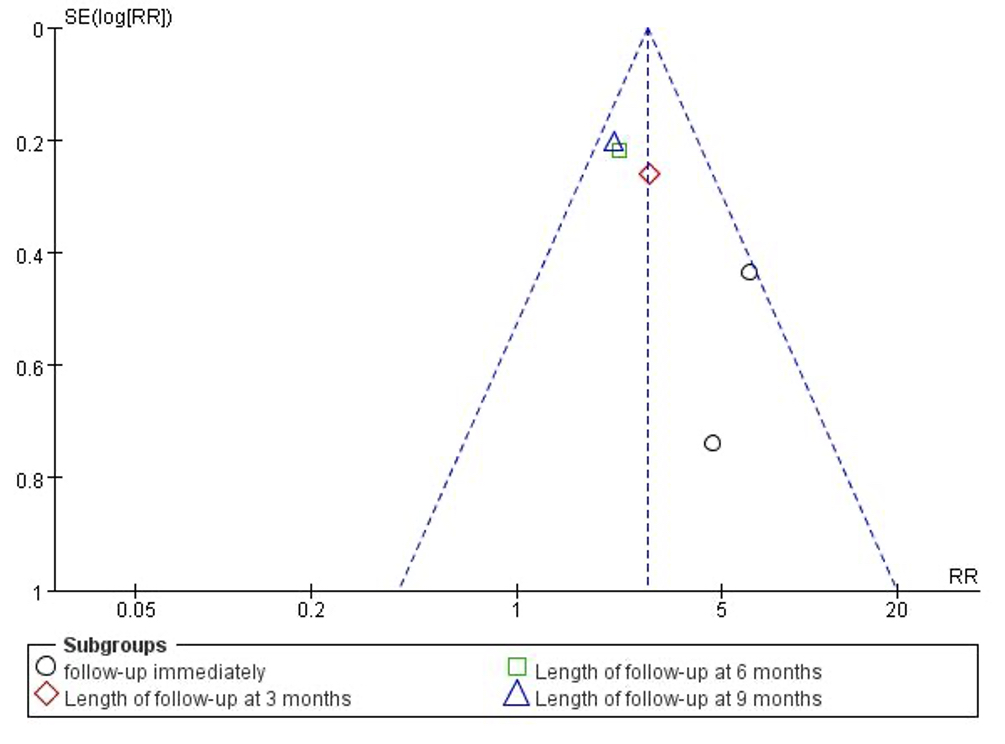

Supplement: Supplementary file 1 [file Image_1.jpg]

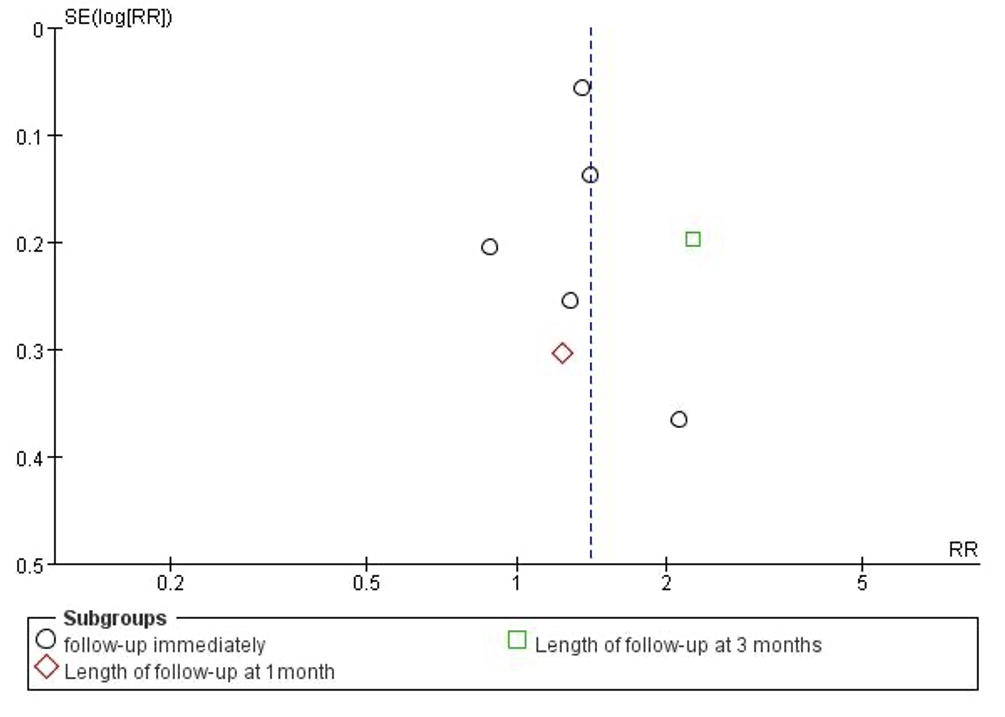

Supplement: Supplementary file 2 [file Image_2.jpg]

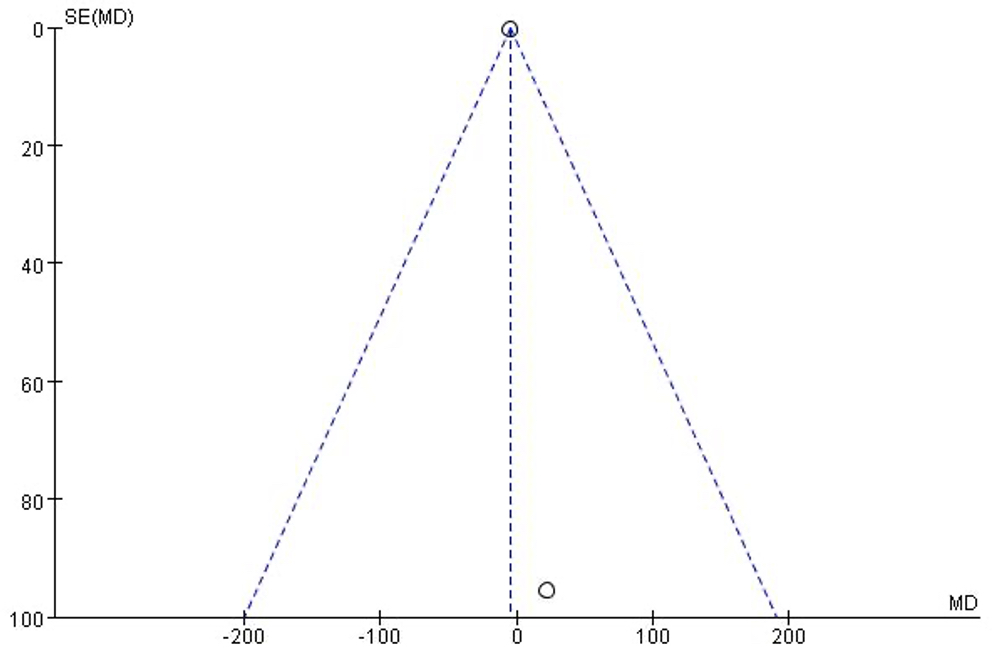

Supplement: Supplementary file 3 [file Image_3.jpg]

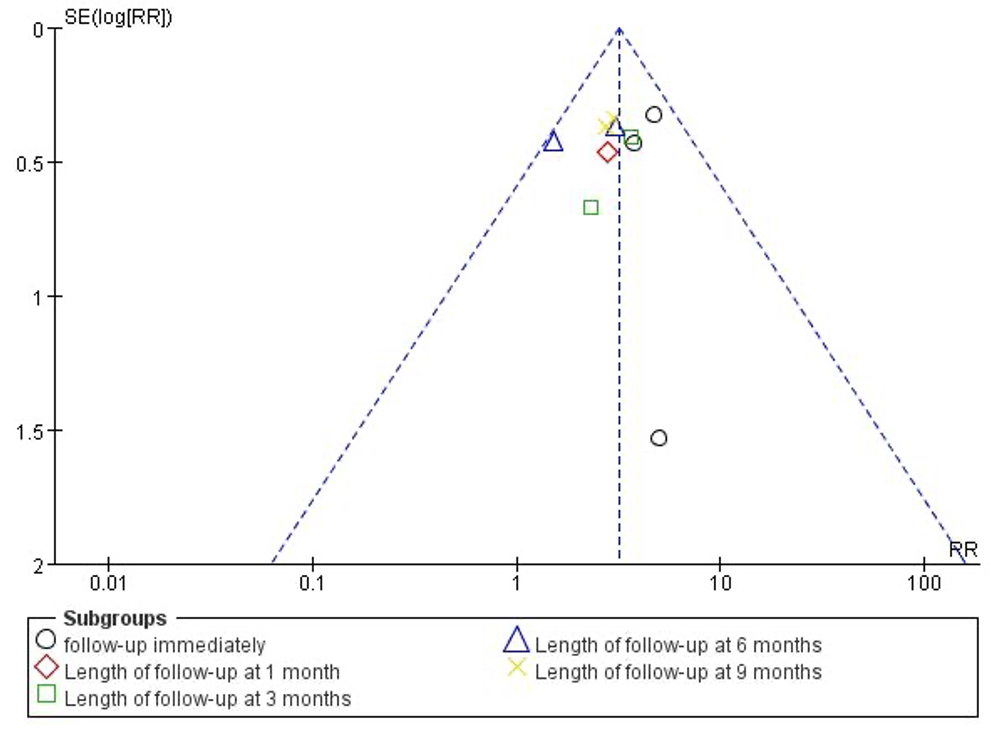

Supplement: Supplementary file 4 [file Image_4.jpg]
